# Gender-Based Violence, Twin Pandemic to COVID-19

**DOI:** 10.1177/0896920520975465

**Published:** 2021-07

**Authors:** Nobuhle Judy Dlamini

**Affiliations:** University of the Witwatersrand, South Africa

**Keywords:** sociology, GBV, COVID-19, gender inequality, intersecting discrimination, racism, pandemic, ageism

## Abstract

The COVID-19 pandemic exposed and exacerbated existing inequalities within countries and across geographies. It reminded us how the world and its people are interconnected. Gender-based violence (GBV), which is an expression of gender inequality and toxic masculinity, is another pandemic that exists in all societies at varying degrees of prevalence and severity.^1^ It requires the same effort and attention that governments globally have given to COVID-19. With half the world under lockdown as governments’ response to COVID-19, GBV increased significantly (UN Women, 2020a). The increase was a reminder of the need to have contingent mitigating mechanism to protect the marginalized, women and girls, against a co-existing pandemic, GBV. The intersection of marginalization and discrimination made certain groups of women more susceptible to GBV and COVID-19 pandemics. These intersecting social identities of vulnerability need equal attention in order to eradicate inequality (Simonovic, 2020).

## Introduction

Gender-based violence (GBV) is violence that is directed against a person on the basis of their sex or gender, and it includes acts that inflict emotional, physical, mental or sexual harm or suffering, threats of such acts, coercion and other deprivations of liberty. ^2^ It is psychological, physical and/or sexual violence perpetrated or condoned within the family, the general community or by the state and its institutions.^2^ GBV occurs in all societies, social classes and cultural groups, and it is a global pandemic that affects one in three women in their lifetime, pre-COVID 19 (The World Bank, 2019). It is prevalent throughout the life-cycle stages for women – infancy, girlhood, adolescence, adulthood and old age. The impact of GBV goes beyond the suffering of survivors and their families, and it is estimated that the cost to the economy can go up to 3.7% of some countries’ GDP.^3^ It affects girls and women’s self-esteem and prevents them from realizing their full rights as human beings and as equal citizens. Violence against girls and women undermines countries’ achievements of at least the first six of the eight United Nations Millennium Development Goals (Solotaroff and Pande, 2014). Women and girls experience violence in a variety of different contexts, within family, the community and broader society. Advancement in technology has added cyberviolence against women as another channel for GBV.

*Pre-COVID-19 Statistics (World Bank, 2019)*:

- **35% of women worldwide** have experienced either physical and/or sexual intimate partner violence or non-partner sexual violence.
- **Globally, 7% of women** have been sexually assaulted by someone other than a partner.
- Globally, as many as **38% of murders** of women are committed by an intimate partner.

Almost 18% of women and girls aged 15–49 years who have ever been in a relationship have experienced physical or sexual violence by an intimate partner in the previous 12 months (United Nations Office on Drugs and Crime (UNODC), 2018).

In 2017, 87,000 women were intentionally killed. The majority of these killings were committed by an intimate partner or a family member (UN Women, 2020a).

- Less than 40% of women who experience violence report it; most of those who do, seek help from a family or a friend; less than 10% of women who seek help go to the police (UN Women, 2020a).
- 200 million women have experienced female genital mutilation/cutting.
- Before COVID-19, 1 in 10 women in the European Union reported having experienced cyber-harassment since the age of 15, including having received unwanted, offensive and sexually explicit emails or SMS messages, or offensive, inappropriate advances on social networking sites.
- According to a November 2016 survey by the All-China Women’s Federation, 30% of married Chinese women had experienced some form of domestic violence (Allen-Ebrahimian, 2020).
- Global cost of violence against women and girls, prior to the pandemic, stood at US$1.5trillion, approximately 2% of global domestic product (UN Women, 2020a, 2020b).

Many studies have shown that children growing up with violence are more likely to become perpetrators of violence in the future or survivors of GBV.

A study on South Asia shows concerning statistics (Solotaroff and Pande, 2014):

- South Asia has the highest levels of excess female child mortality among world regions. Within South Asia, India has the greatest excess female child mortality of all countries for which data are available; Bangladesh, Nepal and Pakistan also show high levels. Since the early 1990s, however, excess female child mortality has declined in Nepal and Sri Lanka and dramatically in Bangladesh. Excess female child mortality in India, however, has remained firmly and largely unchanged.
- South Asia also has the highest rate of child marriage in the world, with 46% of girls married by age 18. In Bangladesh, more than 40% of girls are married by age 15.
- Demographic Health Surveys data from countries whose surveys include questions about physical domestic violence show that almost one-half of surveyed married women in Bangladesh, one-third in India and one-quarter or more in Nepal and Pakistan report physical spousal violence.
- In spite of all eight countries in South Asia having specific constitutional provisions that seek to address gender equality and having signed the Convention on the Elimination of All Forms of Discrimination Against Women, women and girls are still seen as men’s property, they are not seen as equal human beings with equal rights as men and boys.

## Impact of COVID-19 on Women and Girls

The social and economic stress brought by COVID-19 pandemic exacerbated pre-existing toxic social norms and gender inequality. At the time when half of the world population was in lockdown due to COVID-19, the number of women and girls between ages of 15 and 49 who had been subjected to sexual and/or physical violence perpetrated by an intimate partner (GBV) was no less than 243 million (UN Women, 2020a). Many countries from developing and developed economies reported an escalation of GBV during the lockdown; France reported an increase of 30% of domestic violence cases since the lockdown on March 17; Cyprus and Singapore reported an increase in helpline calls by 30% and 33%, respectively, in Argentina emergency calls for domestic violence cases increased by 25% since the beginning of the lockdown; in Canada, Germany, Spain, the UK and the USA, government authorities, women’s rights activists and civil society partners have indicated increasing reports of domestic violence during the crisis, and/or increased demand for emergency shelter (UN Women, 2020a).

According to UN Women (2020a) essential services have experienced increased pressure from the escalation in GBV; In Australia, A Women’s Safety New South Wales survey revealed that 40% of frontline workers reported requests for help by survivors, and 70% reported that the cases received have increased in their level of complexity during the COVID-19 outbreak. There have been increased reports of both physical and verbal attacks on healthcare workers in China, Italy and Singapore (UN Women, 2020a). Simonovic of the Human Rights Watch identified an intersection of marginalization and discrimination making certain groups of women more susceptible to GBV, namely, domestic workers; older women; women with disabilities, including in institutional settings; women without access to technology; women facing overlapping discrimination or inequalities; and women facing housing precarity and violence (Simonovic, 2020):

- The added burden for **domestic workers** varies from exclusion from the safety nets that protect other frontline workers to increased workload with children at the house and employers working from home. In some instances, emotional abuse with no recourse to the perpetrator.
- **Older women** are exposed to abuse by partners, adult children, caregivers and/or other family with whom they live. In spite of the vulnerability of older women, some governments do not consistently collect comprehensive data on violence against older women. For example, the Crime Survey of England and Wales does not collect domestic abuse data on people over 74 years of age. Human Rights Watch’s report to the UN states that the US-based and funded, Demographic Health Survey administered in more than 90 countries does not collect data on violence against women over 49, including during the COVID-19 pandemic, despite the fact that 21.8% of women worldwide are currently over 49. Recognizing that women live on average to 74 years, this practice ignores at least a third of the average woman’s life, an important third in the woman’s life.
- **People with disabilities** living in institutions, including women, have documented abusive treatment and poor conditions in private and state institutions in Brazil, Croatia, Ghana, India, Indonesia, Kazakhstan, Serbia and Somaliland. Restriction of visits during COVID-19 lockdowns has meant that fewer people, especially family members, are able to spot abuse and neglect, including GBV, against women and girls in these facilities.
- **The significant digital gender divide globally** was further exposed by the COVID-19 pandemic. According to the International Telecommunications Union, in 2019, 48% of women used the internet globally compared to 58% of men; the gap grew between 2013 and 2019 in Arab states, Asia, the Pacific and Africa. The UN Broadband Commission in a study across 10 countries in Africa, Asia and South America found women were 30%–50% less likely than men to use the internet to participate in public life. Mobile phones are the primary means of accessing the internet in low- and middle-income countries. According to the GSMA, a global network of mobile operators, 48% of women in these countries use mobile internet; they estimate that a gender gap of 23% persists, representing 313 million fewer women using mobile internet than men. Lack of access to connectivity deprives women the ability to work from home and access critical services like survivor support groups, counselling, health information (including sexual and reproductive health), and other online resources that can be critical lifelines to women experiencing GBV during the lockdown. Those women with digital access have other challenges: an escalation of online GBV, inhibiting or preventing women internet access. Targeted groups include young women, women belonging to ethnic or racial minorities; Indigenous women; lesbian, bisexual and transgender women; women with disabilities; women human rights defenders, journalists; bloggers; women from marginalized groups; and those facing multiple and intersecting forms of discrimination may be particularly and acutely effected by online GBV. According to UN Women (2020a) before COVID-19, 1 in 10 women in European Union reported having experienced cyber-harassment since the age of 15. Internet shutdown by some governments poses another challenge, especially for women because of power differential within societies. Quite a few governments use internet shut down as a form of censorship, and these include Bangladesh, India, Myanmar and Pakistan. Such shutdowns violate multiple rights and can be deadly during a crisis like COVID-19.
- **Women with intersecting discriminations** and/or inequalities are more vulnerable. In many countries, women from already **marginalized communities** find themselves on the frontline as essential workers, in more economic precarity as informal workers, or facing unaddressed institutional racism and health disparities. This includes the so-called BAME community in the UK, which stands for black, Asian and minority ethnic groups, a term that is rejected by some. Migrant and BAME women face exclusion at different levels including language, where only English is used to communicate important information, to lack of women shelters for migrant and BAME women. As an example, as of May 2019, Women’s Aid found that there were only 418 dedicated shelter spaces across England for BAME women, 4 dedicated spaces for women over 45, 12 for women with learning disabilities, and none for deaf women. Migrant women in the UK are in a more precarious situation with abusers using their status to prevent them from seeking help; those on visas such as spousal or fiancé visas have ‘no recourse to public funds’ under the Immigration and Asylum Act 1999, making them ineligible for most government benefits. In Mexico, the government cut the budget by 75% for Casas de la Mujer Indigena (Indigenous Women’s Centre), which service rural and Indigenous areas of Mexico, forcing many of the centers to close leaving rural Indigenous women with nowhere to go. A survey of more than 3000 adults in Colombia found that while nearly 1 in 3 women reported experiencing violence in the home during the pandemic, a slightly higher percentage of healthcare workers did. What is more concerning is that, 45% of people who identify as gender non-conforming experienced violence.

Globally women tend to take on a disproportionate share of the caregiving for children unable to attend day-care, preschool or school due to COVID-19 taking care of the elders amongst other unpaid essential work. This compromises time left for women to participate in income-generating activities.

Other challenges are based on **gender discriminatory legislation**, for instance, in countries where inheritance laws discriminate against women and girls or where same-sex partnerships are not legally recognized, the death of a spouse or father from COVID-19 could have gendered impacts on women, girls and people in same-sex relationships who may lose access to the deceased’s estate, as well as their share of the marital property or inheritance. According to the Mor (2018), out of 161 countries surveyed, only 37 had specific laws granting equal rights for men and women to own, use and control land. Extending land rights and ownership to women, in her view, is one of the prerequisites for achieving the 2030 Agenda for Sustainable Development. Quite a few countries had challenges with places of shelter for displaced GBV survivors, prior to the pandemic. The challenge was exacerbated with the escalation of violence against women. Crises exacerbate existing inequalities; this was learnt from the Ebola and the Zika health crises. Women are disproportionately responsible for unpaid work, informal and low-paying jobs with less security. Job losses for vulnerable women who are in abusive relationships will make it difficult for them to escape. Some pay the ultimate price, killed by an intimate partner.

## Proposed Solutions to GBV During and Post COVID-19

GBV is complex in its causality and requires a multiprong and multi-stakeholder solution.

### Actions by Governments

Gender inequality is one of the main drivers of GBV. Inequality causes harm to its victims and society at large. Addressing this human right violations is the first step to protect society from the two pandemics, health crises and GBV. Economies that do not treat the majority of its citizens as equal citizens, women and girls, deprive all society prosperity and human dignity. Governments have to use policy and funds allocation to empower women economically, ensure they have equal access to land, quality education, health, proper sanitation and safety.

### Gender Budgeting^4^

Gender budgeting is an initiative to use fiscal policy and administration to address gender inequality and women’s advancement. The Council of Europe defines gender budgeting as a ‘gender-based assessment of budgets incorporating a gender perspective at all levels of the budgetary process and restructuring revenues and expenditures in order to promote gender equality’. The ‘Beijing Platform for Action’, adopted in 1995 at the Fourth International Conference for Women, explicitly called for gender budgeting as one way of securing equality between men and women. According to Stotsky et al. (2016), Sub-Saharan African countries were among the earliest countries in the world to adopt gender budgeting with mixed success. South Africa was the first adopter in Sub-Saharan Africa. Some success has been achieved by South Africa, Rwanda and Uganda. Leadership by the finance ministry and input from other stakeholders, namely nongovernmental organizations and donors, are some of the key success factors. The importance of leadership was demonstrated with the waning of gender budgeting efforts in South Africa with changes in parliamentarians (Stotsky et al., 2016).

*UN Women (2020b) has a few recommendations for governments to address the increase of violence against women*:

- Allocation of additional resources, including evidence-based measures, to address violence against women and girls in COVID-19 national response plans;
- Strengthen services for women who experience violence during COVID-19;
- Build capacity of key services to improve quality of response;
- Put women at the centre of policy change, solutions and recovery;
- Ensure sex-disaggregated data are collected to understand the impact of COVID-19 on violence against women and girls to inform the response; and Integration of GBV prevention into women’s socio-economic empowerment initiatives.

### Actions by Civil Society Organizations

Civil society has a major role to play, building strong advocacy and awareness about the GBV scourge. Advocacy work should include bringing all stakeholders together, sensitizing and engaging the private sector on ways to identify and respond to GBV, addressing gender inequality; partnership with media and faith-based leaders for challenging gender stereotypes and toxic masculinity; engage with law enforcement to ensure safety for women and girls. The messaging should be inclusive in terms of language and accessible in terms of media channels used to ensure reaching all vulnerable groups. Collaboration among women organizations, men’s organizations and disability organizations will give birth to a platform where inclusive solutions are co-created.

Different countries have instituted different innovative initiatives to address the increase in gender-based violence; in Spain women are exempt from the lockdown if they experience domestic violence; in Italy prosecutors ruled that perpetrator should leave the family home as opposed to the survivor; in the Canary Islands, Spain women use a code ‘Mask-19’ to alert the pharmacist of domestic violence, which brings police to offer support. ^5^

## The Case of South Africa (ISC-GBVF, 2020)

South Africa is said to have the highest statistics of GBV in the world, including rape and domestic violence (Onyejekwe, 2004). In an article on GBV, South Africa’s second pandemic (Ellis, 2020), President Ramaphosa described the femicide and GBV scourge, stating that, one woman is killed every 3 hours. The COVID-19 pandemic and the lockdown were associated with a spike in GBV and femicide. GBV and femicide are twin pandemics to the COVID-19 pandemic. When the democratically elected government took over in 1994, women represented less than 2% of parliamentarians (20-Year Review SA 1994–2014). Targeted interventions and policy reforms were introduced intended to achieve gender equality. Policies to protect women rights like The Promotion of Equality and Prevention of Unfair Discrimination Act and the Promotion of Administrative Justice Act; The Employment Equity Act of 1998 to facilitate equity in access to formal employment for women across race; The National Crime Prevention Strategy of 1996 making violence against women and children a national priority are amongst many other women-friendly policies. In spite of these initiatives, GBV and femicide remain very high in South Africa. In 2018, when President Cyril Ramaphosa had just assumed office, there was an outcry at the escalation of femicide and violence against women and children. Local women groups and some men groups staged a protest, which was led by the #Totalshutdown Movement. The response was a Presidential Summit where government, civil society and non-governmental organizations came together to strategize on ways to eradicate violence against women. The product was the setting up of the Interim Steering Committee on GBV and Femicide (ISC-GBVF) (2020), which oversaw the development of the Emergency Response Action Plan (ERAP) with an implementation period of six months and total budget allocation of R1.6 billion. The ERAP seeks to mobilize all sectors of society against GBV and to guide the coordination of the national effort. The plan addresses five key intervention areas.

### Five key intervention areas: (International Steering Committee-GBVF, 2020)

- Access to justice for victims and survivors;
- Change norms and behaviour through high-level prevention efforts;
- Urgently respond to victims and survivors of GBV;
- Strengthen accountability and architecture to respond to the scourge of GBVF adequately; and
- Prioritize interventions that facilitate economic opportunities for addressing women’s economic vulnerability.

This initiative was inclusive with collaboration across government departments and civil society. Each area of intervention has human and capital resources committed. The ERAP was delivered in the midst of the COVID-19 pandemic. Eradication of GBV and femicide goes hand-in-hand with eradication of poverty and inequality. In order to achieve the desired change, commitment from all stakeholders and leadership are key.

## Conclusion

GBV and COVID-19 are twin pandemics in South Africa and many other countries to varying degrees. Vulnerable groups, especially women, bear the brunt of health crises and/or any conflict. The empowerment of women, especially those with co-existing social identities of disadvantage, namely race, social class, ageism, disability and sexual orientation, is key for economic prosperity of countries. The COVID-19 pandemic was a reminder, to all countries, for the need to eradicate inequality. It was a reminder to humankind our interconnectedness. It is hoped that we never go back to the pre-COVID anomaly.

## Notes

1. Wealth Health Organisation (2009) *Promoting gender equality to prevent violence against women.* World Health Organisation. Available at: https://apps.who.int/iris/handle/10665/44098 (accessed 25 September 2020).

2. The United Nations’ Office of the High *Commissioner* for Human Rights’ Committee on the Elimination of Discrimination against Women *(1994) Declaration on the Elimination of Violence against Women Proclaimed by General Assembly resolution 48/104 of 20 December 1993* Available at: https://www.ohchr.org/EN/Issues/Women/WRGS/Pages/VAW.aspx (accessed 1 August 2020).

3. The World Bank (2019) *Gender-Based Violence (Violence Against Women and Girls).* Available at: *https://www.worldbank.org/en/topic/socialsustainability/brief/violence-against-women-and-girls* (accessed 14 September 2020).

4. Coovadia Z and Kaplan S (2017*)* Gender budgeting: A tool for achieving equality. *Gender and the Economy*. Available at: https://www.gendereconomy.org/gender-budgeting-a-tool-for-achieving-equality/ (accessed 7 September 2020).

5. Higgins N (Executive Producer) (2020) *Coronavirus: When home gets violent under lockdown in Europe* [BBC Television broadcast]. Brussels. Available at: https://www.bbc.com/news/world-europe-52216966 (accessed 5 October 2020).
